# Non-Cell-Autonomous Regulation of Optic Nerve Regeneration by Amacrine Cells

**DOI:** 10.3389/fncel.2021.666798

**Published:** 2021-04-16

**Authors:** Elena G. Sergeeva, Paul A. Rosenberg, Larry I. Benowitz

**Affiliations:** ^1^Department of Neurology, Boston Children’s Hospital, Boston, MA, United States; ^2^Kirby Center for Neuroscience, Boston Children’s Hospital, Boston, MA, United States; ^3^Department of Neurology, Harvard Medical School, Boston, MA, United States; ^4^Laboratories for Neuroscience Research in Neurosurgery, Boston Children’s Hospital, Boston, MA, United States; ^5^Department of Neurosurgery, Boston Children’s Hospital, Boston, MA, United States; ^6^Department of Neurosurgery, Harvard Medical School, Boston, MA, United States; ^7^Department of Ophthalmology, Harvard Medical School, Boston, MA, United States

**Keywords:** axon regeneration, non-cell-autonomous, optic nerve, amacrine cells, retinal ganglion cells

## Abstract

Visual information is conveyed from the eye to the brain through the axons of retinal ganglion cells (RGCs) that course through the optic nerve and synapse onto neurons in multiple subcortical visual relay areas. RGCs cannot regenerate their axons once they are damaged, similar to most mature neurons in the central nervous system (CNS), and soon undergo cell death. These phenomena of neurodegeneration and regenerative failure are widely viewed as being determined by cell-intrinsic mechanisms within RGCs or to be influenced by the extracellular environment, including glial or inflammatory cells. However, a new concept is emerging that the death or survival of RGCs and their ability to regenerate axons are also influenced by the complex circuitry of the retina and that the activation of a multicellular signaling cascade involving changes in inhibitory interneurons – the amacrine cells (AC) – contributes to the fate of RGCs. Here, we review our current understanding of the role that interneurons play in cell survival and axon regeneration after optic nerve injury.

## Introduction: Factors Determining Retinal Ganglion Cell Survival and Axon Regeneration

A major question in neuroscience is why some neurons in the mature CNS die soon after axonal injury and why almost no neurons are able to regenerate their axons within the CNS even if the cells survive. In a widely studied model of CNS injury and cell death, optic nerve crush (ONC) results in a rapid, transient Ca^2+^ influx into damaged axons from the extracellular space (Knoferle et al., 2010; Vargas et al., 2015; Ribas et al., 2017), followed by early cytoskeletal disruption (Zhai et al., 2003; Beirowski et al., 2010) and autophagy-mediated disintegration of the axons (Komatsu et al., 2007; Beirowski et al., 2010; Knoferle et al., 2010), which results in continuous degeneration of axons distal to the injury site (McKeon et al., 1995; Beirowski et al., 2010; Knoferle et al., 2010; Anderson et al., 2016). Ca^2+^ influx along with injury signals propagating retrogradely from the axonal stump activate a MAP kinase signaling cascade involving dual-leucine kinase (DLK), leucine-zipper kinase (LZK), and their downstream effectors that culminates in RGC death (Kikuchi et al., 2000; Knoferle et al., 2010; Fernandes et al., 2012; Katome et al., 2013; Watkins et al., 2013; Welsbie et al., 2013; Vargas et al., 2015; Yang et al., 2015; Ribas et al., 2017); at the same time, activation of SARM1 culminates in axon degeneration (Gerdts et al., 2016). Certain types of RGCs, specifically intrinsically photosensitive RGCs and alpha-RGCs, are relatively resilient, although in the absence of treatment, most RGCs will eventually die (Park et al., 2008; Duan et al., 2015; Norsworthy et al., 2017; Tran et al., 2019).

While strategies to counteract the pathways leading to cell death can improve RGC survival, the effects are often transitory, or allow long-term survival in a compromised state, or even suppress regeneration (Janssen et al., 2013; Katome et al., 2013; Watkins et al., 2013; Welsbie et al., 2013; Ribas et al., 2017). For example, RGC death following ONC can be suppressed by deletion of the pro-apoptotic regulator bcl2-associated X protein BAX, but this does not improve axon regeneration (Donahue et al., 2020). Inhibition of DLK and LZK has a robust effect on RGC survival but drastically suppresses RGC axon regeneration (Watkins et al., 2013). Deletion of phosphatase and tensin homologue (PTEN) or upregulation of another mTOR enhancer, osteopontin, induces high but transient protection of RGCs and axon regeneration, and most RGCs go on to die after several weeks (Park et al., 2008; Duan et al., 2015; Li et al., 2017a). Although axon regrowth after injury obviously depends on cell survival, the two processes are distinct, and surviving RGCs do not regenerate axons by default (Chierzi et al., 1999; Goldberg and Barres, 2000; Goldberg et al., 2002a). However, in an exciting recent discovery, Patel et al. showed that inhibition of germinal cell kinase IV (GCK-IV) promotes RGC survival without suppressing axon regeneration (Patel et al., 2020).

The low intrinsic capacity of mature neurons to regenerate axons within the CNS is caused in part by developmentally regulated expression of factors that prevent excessive cell growth and sprouting (He and Jin, 2016; Benowitz et al., 2017; Yin et al., 2019). Manipulation of intrinsic growth pathways, such as activating the PI3K/Akt/mTOR pathway by deleting its endogenous repressor PTEN and others induces significant axonal regeneration in injured RGCs (He and Jin, 2016; Benowitz et al., 2017; Yin et al., 2019), as does manipulating developmentally regulated transcription factors that suppress neurons’ growth program (Moore et al., 2009, 2011; Apara et al., 2017; Norsworthy et al., 2017; Galvao et al., 2018; Cheng et al., 2020).

At the same time, many studies demonstrate the importance of the environment surrounding injured axons in suppressing or promoting regeneration. Modulation of extrinsic suppressors of growth such as myelin-associated inhibitors, components of extracellular matrix, microglia, or attenuation of pericyte-derived fibrosis, leads to modest improvements of axon regeneration (Benowitz et al., 2017; Yin et al., 2019). On the other hand, some extrinsic factors can promote regeneration. The latter include resident glia and inflammatory cells, macrophages and neutrophils, that can produce a variety of growth factors and chemokines that promote regeneration and RGC survival, including oncomodulin, SDF-1, and, in response to CNTF gene therapy, CCL5 (Benowitz et al., 2017; Yin et al., 2019; Xie et al., 2021). Combinatorial treatment strategies that overcome cell-extrinsic or cell-intrinsic suppressors of growth while simultaneously activating neurons’ intrinsic growth state result in impressive levels of regeneration (Fischer et al., 2004a,b; Kurimoto et al., 2010; Sun et al., 2011; de Lima et al., 2012; Dickendesher et al., 2012; Wang et al., 2012; Zhang et al., 2019). Other important factors present in the environment of RGCs derive from other neurons and glia, and include (1) the navigational cues provided by cells along the trajectory of developing axons and in visual target areas (e.g., netrins, semaphorins, Ephrins, Wnts, Slits) (Pfeiffenberger et al., 2005; Feldheim and O’Leary, 2010; Varadarajan and Huberman, 2018); (2) cues from neighbor cells that change RGCs’ program of gene expression (Livesey and Cepko, 2001; Goldberg et al., 2002b); (3) regeneration of RGC axons through a peripheral nerve graft (So and Aguayo, 1985; Vidal-Sanz et al., 1987; Aguayo et al., 1991). However, the significance of retinal interneurons and retinal circuitry after RGC axonal injury has received relatively little attention.

A factor that is now coming to light is the instructive role that amacrine cells (AC), the inhibitory interneurons of the retina, play in regulating RGC survival and axon regeneration. ACs either form direct, mostly (but not exclusively) inhibitory synapses (or gap junctions) onto RGC or modulate excitatory inputs from bipolar cells (BC) and inhibitory inputs from other ACs (Kolb and Famiglietti, 1974; Kim et al., 2015). Growing evidence indicates that signaling in this complex circuitry changes upon injury to RGC axons and, in turn, influences RGCs’ ability to survive and regrow their axons. In this review we focus on the emerging role of retinal circuitry, and amacrine cells in particular, in RGC survival and axon regeneration after optic nerve injury.

## Amacrine Cell Activity and Rgc Growth State

The earliest evidence of a circuit-level influence on RGC axon outgrowth came from studies carried out in primary retinal cell cultures. Purified neonatal rat RGCs show an irreversible reduction in axon outgrowth when co-cultured with purified ACs but not when co-cultured with BCs (Goldberg et al., 2002b), suggesting that signals from ACs instruct RGCs to decrease their intrinsic growth ability. This effect was seen using isolated AC membranes, pointing to a contact-mediated suppression of RGCs’ growth capacity (Goldberg et al., 2002b; Goldberg, 2004). The loss of RGCs’ ability to elongate axons coincides temporally with a period of enhanced dendritic growth, suggesting that RGCs can be either in a primarily axonal or dendritic growth state, and that their intrinsic growth state is switched developmentally by a signal arising from ACs (Goldberg et al., 2002b; Goldberg, 2004). *In vivo*, the decline in RGCs’ growth state is associated with numerous changes in these cells’ program of gene expression, including an upregulation of the growth suppressive Kruppel-like transcription factors Klf-4 and Klf-9, and down-regulation of the growth-promoting transcription factors Klf-6 and Klf-7 (Moore et al., 2009, 2011; Apara and Goldberg, 2014). The developmentally regulated suppressor of axonal growth, PTEN, also shows increased expression during this transition (Sakagami et al., 2012). In turn, mTOR decreases in expression during development and is downregulated even more after axonal injury, thereby diminishing RGCs’ regenerative capacity (Park et al., 2008; Belin et al., 2015). The JAK2/STAT3 pathway can promote regeneration when activated by certain cytokines, e.g., CNTF, LIF, or IL6, although in the adult CNS, this signaling is negatively regulated by SOCS3 (Smith et al., 2009). In mature mice, recombinant CNTF has little effect on RGCs whereas CNTF gene therapy promotes considerable optic nerve regeneration through an indirect mechanism that involves activation of innate immune cells and glia and expression of chemokine CCL5 (Xie et al., 2021). Other developmentally and injury-regulated intrinsic factors continue to be discovered (He and Jin, 2016), although a direct link between these changes and RGC-amacrine cell contact has not yet been investigated.

The growth state of RGCs can be altered by their level of physiological activity, and ACs play an important role in this regard (Goldberg et al., 2002a; Goldberg, 2012; Li et al., 2016; Zhang et al., 2019). In culture, a weak, physiological level of current applied to purified rat primary RGCs, or membrane depolarization by elevated extracellular potassium, improves BDNF-induced axon outgrowth (Goldberg et al., 2002b). *In vivo*, diminished physiological activity in RGCs diminishes these cells’ capacity to regenerate axons and this decline can be partially reversed by expressing melanopsin in RGCs and exposing to light (inducing activation of cell-intrinsic growth pathway mTOR) (Li et al., 2016), by expressing a depolarizing receptor and applying its ligand, or by increasing RGC neural activity with patterned visual stimulation (Lim et al., 2016).

**FIGURE 1:**
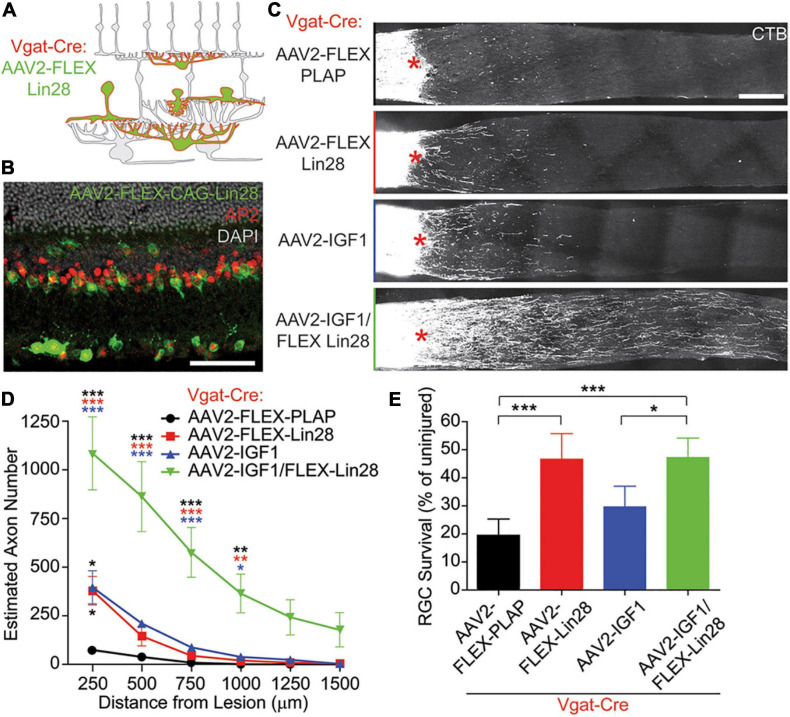
Lin28 expression in inhibitory neurons promotes RGC survival and IGF1-induced axonal regeneration. **(A,B)** Schematic **(A)** and example **(B)** confocal image stack showing expression of AAV2-FLEX-Lin28 in the intact Vgat-Cre transgenic retina where Lin28 expression is restricted to amacrine and horizontal cells. **(C)** Representative confocal image stacks of CTB labeled RGC axons 2 weeks after optic nerve crush with amacrine cell restricted expression of Lin28. Asterisks indicate crush site. **(D)** Quantification of the extent of RGC axon regeneration in treatment groups restricted to amacrine cells. Asterisk colors indicate the group that the p value was significant against. **(E)** Quantification of RGC survival relative to RGC density observed in intact retinas in treatment groups restricted to amacrine cells. *n* = 5 mice per group. Scale bar, 50 μm in **(B)**, and 200 μm in **(C)**. *, **, ***p < 0.05, 0.01, 0.001, respectively. Reprinted from Zhang et al. (2019) with permission.

RGC activity is reduced by the hyperpolarizing inhibitory drive from ACs, suggesting that such inhibition could suppress regeneration *in vivo*; and conversely, activation of RGCs by bipolar cells or via silencing of ACs could be permissive for regeneration. More generally, circuit-level activity levels of the retina can alter the activity state of RGCs and thus influence axon regeneration in the optic nerve. Zhang et al. (2019) showed that optic nerve injury increases the activity of ACs (Zhang et al., 2019), which in turn puts a brake on regeneration by inhibiting RGC activity and reducing these cells’ responsiveness to growth factors (Zhang et al., 2019) (Figure 1). When hyperactive ACs were silenced, as confirmed by diminished levels of the immediate-early gene c-fos in these cells, RGCs showed increased physiological activity and improved responsiveness to insulin-like growth factor IGF1 (Zhang et al., 2019). This improved responsiveness was mediated by increased expression of the IGF1 receptor on RGCs’ primary cilia, which serve as the growth factor-sensing antennae of these cells (Guemez-Gamboa et al., 2014), leading to increased RGC survival and axon regeneration. In this study, AC activity was suppressed by either overexpressing the potassium channel Kir2.1 or by overexpressing an RNA-binding insulin-sensitizing protein Lin28 specifically in ACs and horizontal cells. Importantly, whereas IGF1 overexpression or blocking inhibition by either silencing ACs or suppressing neurotransmission with a cocktail of GABA and glycine receptor antagonists induced a moderate level of regeneration by itself, the combination of AC silencing plus IGF1 overexpression had a strongly synergistic effect via increased RGC activity and IGF1 signaling competence (Zhang et al., 2019). Interestingly, Lin28 overexpression in both RGCs and ACs or only in RGCs induced comparable levels of RGC axon regeneration suggesting that Lin28 also has cell-autonomous effects (Wang et al., 2018; Zhang et al., 2019). Further work will be required to understand how Lin28 expression in ACs is linked to AC activity and how axonal injury in RGCs leads to changes in presynaptic retinal circuitry and AC hyperactivation.

Although RGCs can respond to some growth factors without elevating their physiological activity, such as SDF-1 (Yin et al., 2018) and CCL5 (Xie et al., 2021), their ability to respond to the growth factors BDNF and IGF1 is dependent upon enhanced physiological activity (Goldberg et al., 2002a; Duan et al., 2015; Zhang et al., 2019). Activation of RGCs leads to their depolarization and Ca^2+^ influx which elevates intracellular cAMP levels (Meyer-Franke et al., 1998) and mediates enhanced mTOR signaling and phosphorylation of its downstream effector S6 kinase (Park et al., 2008; Duan et al., 2015; Zhang et al., 2019). Ca^2+^ influx upon depolarization of RGCs can also trigger rapid post-translational modifications, *e.g*. phosphorylation of pre-existing transcription factors such as CREB, SRF/FLK, and MEF2, which in turn drive activity-dependent transcription of immediate-early genes followed by late response genes (Yap and Greenberg, 2018). The activity-regulated genes control the expression of numerous effectors of cell survival and regeneration, including growth factors and receptors to growth factors (Yap and Greenberg, 2018). Conversely, excessive inhibition of RGCs by hyperactive ACs would be expected to result in reduced Ca^2+^ influx into RGCs, diminished Ca^2+^-mediated, activity-dependent transcription, and suppression of RGCs’ intrinsic growth state. However, despite the increased inhibitory drive onto RGCs due to elevated amacrine cell activity after optic nerve damage, elevation of RGCs’ intrinsic growth state (PTEN deletion, SOCS3 deletion combined with CNTF, manipulation of transcription factors) can nevertheless increase axon regeneration, as we discussed above.

## Amacrine Cells and Zinc Signaling in the Retina

**FIGURE 2:**
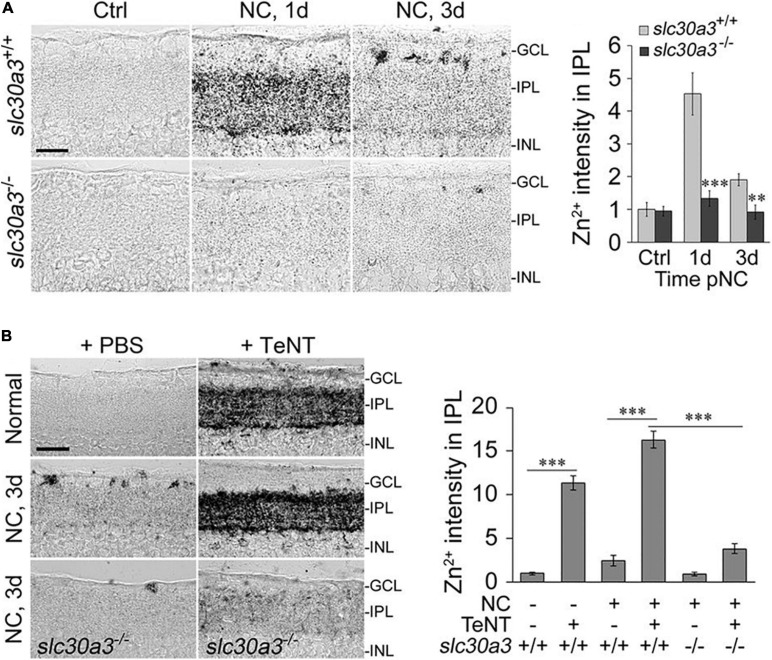
Zn^2+^ accumulation in the retina and its role in axon regeneration after optic nerve crush (NC). **(A)** Zinc accumulates in the inner plexiform layer (IPL) of the retina shortly after NC in wild-type mice (slc30a3^+/+^) but not in mice lacking the zinc transporter ZnT3 (slc30a3^–/–^): images and quantitation of AMG staining in the IPL (*n* = 6 retinas per group) of wild-type and slc30a3^−/−^ littermates. Note elevation of AMG signal on day 1 following NC in wild-type mice and decline to near normal level by day 3 (Scale bar, 25 μm; ^∗∗^*P* < 0.01, ^∗∗∗^*P* < 0.001). **(B)** Tetanus toxin (TeNT) blocks vesicular release of Zn^2+^, causing continued Zn^2+^ build-up in the IPL: images and quantification of AMG staining in the IPL after NC with and without intraocular injection of TeNT (20 nM). Note elevation of AMG staining in the IPL of normal, uninjured mice and in wild-type mice, at 3 days after NC, a time point at which AMG staining in the IPL would normally dissipate. Deletion of the gene encoding ZnT3 eliminates Zn^2+^ accumulation in the IPL (Scale bar, 50 μm; ^∗∗∗^*P* < 0.001). Adapted from Li et al. (2017a) with permission.

In addition to diminishing RGCs’ activity state, do ACs produce other signals that suppress RGC survival and regenerative ability? Our lab recently reported that one such signal may be mobile zinc (Zn^2+^) (Li et al., 2017a; Trakhtenberg et al., 2018). Elevation of mobile Zn^2+^ in AC terminals within the inner plexiform layer (IPL) of the retina, as demonstrated by selenite autometallography (AMG), is one of the earliest changes seen in mouse retina after optic nerve injury (Li et al., 2017a) (Figure 2).

Normally, zinc is covalently bound to proteins, including many transcription factors and enzymes, enabling their folding and thus their functionality (McCall et al., 2000; Kochanczyk et al., 2015). Some neurons, including particular cells in the hippocampus, cerebral cortex, and spinal cord, sequester Zn^2+^ in synaptic vesicles and co-release it with classical neurotransmitters (Nakashima and Dyck, 2009; Sensi et al., 2009, 2011; Pan et al., 2011; Kimura and Kambe, 2016). Intracellular levels of mobile Zn^2+^ can vary depending on many factors, including oxidative stress and liberation of Zn^2+^ from oxidized proteins (Aravindakumar et al., 1999; Sensi et al., 1999; Spahl et al., 2003; Aras and Aizenman, 2011), redistribution of Zn^2+^ between intracellular pools (Sekler et al., 2007; Maret, 2017; Ji et al., 2020), and transcriptional and posttranscriptional regulation of Zn^2+^-regulating proteins (Saydam et al., 2002; Jackson et al., 2008). It is important to maintain Zn^2+^ concentrations within a narrow range in different intracellular compartments to maintain proper Zn^2+^ availability to numerous Zn^2+^-binding proteins while at the same time preventing mismetallation and Zn^2+^ toxicity (Aras and Aizenman, 2011). For this purpose, a complex homeostatic machinery comprised of metal buffering proteins – metallothioneins and zinc transporters (ZnTs and ZIPs) has evolved (Hidalgo et al., 2001; Cousins et al., 2006; McAllister and Dyck, 2017).

Metallothioneins, glutathione and other metal-containing peptides and proteins can liberate Zn^2+^ and copper ions (Cu^+^ or Cu^2+^) when subjected to oxidative stress (Maret, 1995). For example, reactive oxygen species and peroxynitrite can oxidize residues on the metal-binding sites of metal-binding proteins and release the cations (Sensi et al., 1999; Hidalgo et al., 2001; Spahl et al., 2003; Zhang et al., 2004; Aras and Aizenman, 2011). Cu^+^ and Cu^2+^, as redox-active ions, can directly displace Zn^2+^ from metallothioneins or engage in oxidative reactions, ultimately leading to more Zn^2+^ release (Krȩżel and Maret, 2017). The apparent elevation of mobile Zn^2+^ in AC terminals that synapse onto RGCs that occurs soon after optic nerve injury points to AC and Zn^2+^ dysregulation as a potential major factor of abnormal retinal circuit homeostasis after injury (Li et al., 2017a) (Figure 2).

### Role of Nitric Oxide and Presumptive Role of Glutamate and Bipolar Cells in Retinal Zn^2+^ Homeostasis

Little is known about the mechanisms underlying the increase in AMG signal in the retina following the injury of RGC axons. A preliminary report used a novel fluorescent nitric oxide (NO) sensor, Cu_2_FL2E (Pluth et al., 2011), to provide evidence that the production of NO is rapidly and persistently upregulated in the retina after optic nerve injury, and that NO generation is upstream of the accumulation of AMG signal in the retinal IPL (Li et al., 2017b). One possibility is that reactive nitrogen species produced after injury, e.g., peroxynitrite, can liberate Zn^2+^ from metallothioneins (Zhang et al., 2004; Nakamura et al., 2015; Wolhuter et al., 2018). Alternatively, NO can contribute to an increase of intracellular Zn^2+^ via a cGMP/PKG-dependent release of Zn^2+^ from internal stores (Jang et al., 2007).

NO is synthetized by nitric oxide synthetase, one isoform of which, NOS1, is expressed exclusively in a subset of ACs (Yamamoto et al., 1993; Oh et al., 1998). Production of NO in ACs after optic nerve injury points to the existence of an as yet unidentified retrograde signal linking RGC axon injury and NOS1 activation. NOS1 activation can be triggered by Ca^2+^ entering ACs upon activation of voltage-gated calcium channels or through NMDA or AMPA receptors (Christopherson et al., 1999). These latter receptors can be activated by glutamate that is either synaptically released by BCs or elevated due to a reversal of glutamate transporters, e.g., GLT-1, EAAC1, GLAST, that are expressed on retinal neurons or glia, including astrocytes and Mueller cells. Glutamate transporters normally take up extracellular glutamate but can reverse the direction of transport and release glutamate upon changes in Na^+^ and K^+^ gradients or membrane potential (Szatkowski et al., 1990; Danbolt, 2001; Grewer et al., 2008; Armbruster et al., 2016; Rimmele et al., 2017). Our preliminary studies show that BC-specific knockout of GLT-1 may prevent mobile Zn^2+^ accumulation in AC terminals after ONC, as does inhibition of NMDA receptors (Hanovice et al., 2019). Taken together, these results suggest that reversal of the glutamate transporter GLT-1 in BCs, activation of NMDA receptors, and NO elevation may act upstream of Zn^2+^ liberation and accumulation in AC terminals after optic nerve injury (Hanovice et al., 2019).

### Effect of Presynaptic Zinc on Retinal Ganglion Cells

In line with previous studies showing that Zn^2+^ levels in the brain (visualized by AMG) are abolished in mice lacking the zinc transporter protein ZnT3, the accumulation of Zn^2+^ in AC terminals following ONC is similarly absent in ZnT3 knock-out mice (Li et al., 2017a) (Figure 2A). Because ZnT3 enables Zn^2+^ to be sequestered in synaptic vesicles (Palmiter et al., 1996), this finding implies that the Zn^2+^ that is mobilized in ACs after ONC is stored in synaptic vesicles (Palmiter et al., 1996; Li et al., 2017a). In conformity with this idea, the Zn^2+^ that accumulates in the retinal IPL after ONC normally dissipates by 48 hour after ONC (Li et al., 2017a) but continues to accumulate if exocytosis is inhibited using *Clostridium tetani* neurotoxin (TeNT) (Li et al., 2017a; Sergeeva et al., 2019) (Figure 2B). Blockade of synaptic release from AC terminals with TeNT promotes RGC survival and optic nerve regeneration (Li et al., 2017a; Sergeeva et al., 2019). These data suggest that Zn^2+^ packaged into synaptic vesicles and released from AC terminals, or the neurotransmitter used by these neurons, or both, may negatively affect RGC survival and block axon regeneration.

It should be noted, however, that because the chelators used in the aforementioned studies are not entirely specific to Zn^2+^, it remains possible that other cations, e.g., Cu^+^ or Cu^2+^, could also be involved. Copper is stored in synaptic vesicles and released upon depolarization (Kardos et al., 1989). Moreover, the method used to detect Zn^2+^, e.g., AMG, although generally regarded as being specific to Zn^2+^ (Danscher and Stoltenberg, 2005), may also provide ambiguous results, as selenite may potentially form complexes with other divalent cations, suggesting that vesicular Cu^+^/Cu^2+^ may potentially contribute to AMG staining. On the other hand, the observation that the AMG signal in the retinal IPL is abolished in mice lacking ZnT3 supports the hypothesis that the AMG signal reflects Zn^2+^ *per se*, provided that ZnT3 does not transport other divalent cations, such as copper. At this stage, we also do not know whether other metals act downstream or upstream of Zn^2+^ release and accumulation.

Synaptic release of Zn^2+^ from ACs could affect RGC signaling via numerous pathways. Synaptic Zn^2+^ can modulate the activity of NMDA, GABA and glycine receptors, thereby modulating cell excitation and inhibition (Suwa et al., 2001; Kaneda et al., 2005; Sensi et al., 2011; Vergnano et al., 2014). Zn^2+^ modulates glycine receptors in a biphasic manner, potentiating inhibition at low micromolar concentrations while suppressing glycinergic currents at high concentrations (Kaneda et al., 2005). Potentiation of glycine receptors on RGCs would be expected to decrease RGC activity which, as noted above, would diminish RGC survival and axon regeneration (Goldberg et al., 2002a; Goldberg, 2012; Zhang et al., 2019). In addition, Zn^2+^ interacting with the Zn^2+^-sensing receptor ZnR/GPR39 could regulate the transport of Na^+^, K^+^, Cl^–^ (Chorin et al., 2011; Saadi et al., 2012) and trigger Gα_*q*_-dependent signaling and subsequent release of Ca^2+^ from endoplasmic reticulum stores, thereby modulating ERK/MAPK and PI3K/Akt/mTOR signaling, both of which are important for cell survival and growth (Azriel-Tamir et al., 2004; Hershfinkel, 2018). Potentially, intracellular Zn^2+^ elevation can induce cell death by upregulating proapoptotic factors (Jiang et al., 2001; Zhang et al., 2004, 2006, 2007; Cohen et al., 2012), mitochondrial impairment (Sensi et al., 1999; Baud et al., 2004; Ji et al., 2020), synthesis of reactive oxygen species (Wang et al., 2004; Bishop et al., 2007), activation of MAPK/p38 signaling and activation of voltage-gated K^+^ channels, leading to K^+^ efflux (McLaughlin et al., 2001; Bossy-Wetzel et al., 2004; Zhang and Rosenberg, 2004; Zhang et al., 2004, 2006, 2007; McCord and Aizenman, 2013).

Chelation of Zn^2+^ after ONC can potentially inhibit histone deacetylases (HDACs), enzymes that deacetylate histone proteins, thereby rendering chromatin more accessible for transcription. The deacetylating activity of HDACs depends on the binding of Zn^2+^ in the HDAC active site pocket (Pelzel et al., 2010; Li et al., 2019). Prevention of histone deacetylation by inhibition of HDAC activity caused by removal of Zn^2+^ from HDACs can potentially facilitate transcription of activity-dependent genes and ultimately add to the effects of RGC activation. Along these lines, inhibition of HDAC activity alone has been shown to protect RGCs after injury (Gaub et al., 2011; Zhang et al., 2012; Chindasub et al., 2013; Janssen et al., 2013; Schmitt et al., 2014).

An additive effect on RGC survival by metal chelators was observed in combinatorial treatment with deletion of PTEN, producing survival of RGCs that was substantially greater at 12 weeks post ONC compared to PTEN deletion itself (Li et al., 2017a). Knockdown of another intrinsic suppressor of axonal growth, Klf-9, also demonstrated enhanced RGC survival when combined with chelation (Trakhtenberg et al., 2018).

## Why Do Amacrine Cells Become Hyperactive? A Hypothesis

It is largely unknown how or why ACs become hyperactive after optic nerve injury (Zhang et al., 2019). Activation of NMDA or AMPA receptors on ACs by glutamate released from BCs leads to AC depolarization, increased firing, and increased release of glycine and GABA onto RGCs, as well as onto BCs and other ACs (Kolb and Famiglietti, 1974). Activation of GABA or glycine receptors on ACs by GABA or glycine released from other ACs causes these cells to become hyperpolarized to a level closer to the reversal potential for Cl^–^, which in these cells is normally more negative than the membrane potential, reducing action potential firing, with a net effect of decreasing inhibitory tone projecting onto RGCs. However, under some circumstances, for example, early in development, the reversal potential for chloride may be depolarized with respect to the membrane potential due to the electrochemical gradient driven by high intracellular Cl^–^. Such switch in GABA function is mediated by changes in expression or localization of Cl^–^ transporters: the neuron-specific K^+^- Cl^–^ cotransporter KCC2 and the Na^+^ - K^+^- Cl^–^ cotransporter NKCC1 expressed in immature neurons (Kaila et al., 2014). Consequently, the activation state of ACs depends not only on the sum of excitatory and inhibitory inputs onto these cells at any moment, but also on the Cl^–^ gradient that determines the polarity of the GABAergic and glycinergic drive onto these cells. Alteration of the Cl^–^ gradient may be important in retinal network dysfunction and has been investigated in several studies (Hoffpauir et al., 2006; Krishnan and Gleason, 2015).

Cl^–^ gradient alteration induced by decreased KCC2 function or expression is an important cause of disinhibition in cells and circuits, and has been shown to participate in several neurological disorders including epilepsy (Moore et al., 2017; Liu et al., 2019), spasticity after spinal cord injury (Boulenguez et al., 2010; Chen et al., 2018), autism and Rett syndrome (Tang et al., 2016) and chronic pain (Coull et al., 2003; Hasbargen et al., 2010), all of which are characterized by a failure of inhibition and neural hyperactivation (Nabekura et al., 2002; Kaila et al., 2014). KCC2 cotransport utilizes a K^+^ gradient to extrude Cl^–^ (Payne et al., 2003; Kaila et al., 2014), therefore the Cl^–^ transporter activity may decrease with high extracellular K^+^ following ischemia, injury, Na^+^/K^+^ ATP dysfunction and reduced production of ATP due to mitochondrial compromise (Kleber, 1984; Hughes and Cidlowski, 1999; Kaila et al., 2014; Doyon et al., 2016). KCC2 is highly expressed in the retina (Vardi et al., 2000; Vu et al., 2000). With increased extracellular K^+^ after K^+^ efflux from injured RGCs (Yu et al., 1997; Diem et al., 2001; Zhong et al., 2013) or activated microglia (Fordyce et al., 2005) KCC2-dependent Cl^–^ extrusion in ACs may be diminished.

KCC2, like many intracellular proteins, can be regulated by phosphorylation, trafficking and proteolytic cleavage (Kaila et al., 2014; Doyon et al., 2016; Kahle and Delpire, 2016). Extracellular modifiers of KCC2 expression and function include BDNF/TrkB, serotonin/5HT2A, glutamate/NMDA, the Zn^2+^ - sensing receptor GPR39, and noradrenaline signaling (Wake et al., 2007; Hershfinkel et al., 2009; Bos et al., 2013; Watanabe and Fukuda, 2015; Tang et al., 2019). In addition, KCC2 activity can be suppressed by both NO and intracellular zinc (Yassin et al., 2014) shown to be elevated in ACs after injury (Li et al., 2017a,b).

KCC2 independent-, NO-mediated elevation of intracellular Cl^–^ could be another potential mechanism of AC disinhibition after optic nerve injury. In chick ACs *in vitro*, NO transiently reverses GABA- and glycine-gated currents, converting inhibition of ACs into excitation, thereby increasing the firing of these cells and thus enhanced inhibitory drive on their synaptic partners (e.g., RGCs). This NO-induced shift in E_*Cl–*_ is likely due to release of Cl^–^ from intracellular stores (Hoffpauir et al., 2006; Krishnan and Gleason, 2015; Krishnan et al., 2017; Maddox and Gleason, 2017; Maddox et al., 2018). In addition, NO may drive synaptic glutamate release from BCs without membrane depolarization via a TRPC Ca^2+^ influx-mediated pathway, as shown in the chick retina (Maddox et al., 2018), further depolarizing ACs.

In summary, dysregulation of Cl^–^ gradients in the inner retina may be a part of the early pathological process following optic nerve or RGC injury. Reciprocally connected ACs and BCs, in the face of Cl^–^ gradient collapse, can form circuits with positive feedback loops that may rapidly lead to hyperactivation of ACs and thus increased inhibition of their synaptic targets (Marc and Liu, 2000; Marc et al., 2014; Doyon et al., 2015, 2016). Whether the complex retinal circuitry is particularly susceptible to persistent disinhibition of ACs after injury remains to be studied.

## Conclusion: From Retinal Ganglion Cells to Retinal Circuits

Silencing ACs or introducing chelators into the eye to suppress Zn^2+^ accumulation in amacrine cell terminals are additive with the effects of manipulating RGC-intrinsic factors (PTEN deletion, Klf-9 suppression, upregulation of osteopontin) on RGC survival and regeneration (Li et al., 2017a; Trakhtenberg et al., 2018; Zhang et al., 2019). These findings suggest that dysfunction of the retinal network, and particularly interneuron (AC) dysfunction, is part of the pathological process following optic nerve injury, and that the capacity of RGCs to survive and regenerate may depend in part on the activity of the other retinal neurons with which they are connected.

Although the non-cell-autonomous regulation of neuronal survival and pathological functioning by other neurons is just starting to be recognized as being important after optic nerve injury, neuronal circuits have been implicated in various pathological processes and cell death in other neurodegenerative diseases (Palop et al., 2006; Simon et al., 2016). In amyotrophic lateral sclerosis, hyperexcitability and death of motoneurons have been attributed to a non-cell autonomous response to a defect in premotor interneurons (Wainger et al., 2014; Held et al., 2019). In Parkinson’s disease, alterations of basal ganglia circuitry have been shown to precede loss of substantia nigra neurons (McGregor and Nelson, 2019), as was shown for striatal spiny neurons in Huntington’s disease (Creus-Muncunill and Ehrlich, 2019). In Alzheimer’s disease, early circuitry dysfunction may be induced by amyloid beta-mediated suppression of glutamate reuptake and a consequent vicious cycle of neuronal hyperactivation and cell death (Zott et al., 2018, 2019). In autism and Alzheimer’s disease, dysfunction of interneurons has been implicated (Palop et al., 2006; Palop and Mucke, 2016; Martinez-Losa et al., 2018). Disruption of excitatory and inhibitory circuits and excitatory-inhibitory imbalance also seem important in the pathogenesis of Rett syndrome and autism (Nelson and Valakh, 2015; Patrizi et al., 2020). Here we assemble evidence that optic nerve injury induces changes in retinal circuitry that is initiated by an as-yet unidentified signal from injured RGCs to retinal interneurons that alters the function of amacrine cells, in turn influencing the survival and regenerative capacity of injured RGCs.

Despite considerable progress in the areas of RGC protection and optic nerve regeneration, there is still a long way to go before we achieve satisfactory levels of functional recovery. One factor that is now coming to be appreciated is the crosstalk between cell-intrinsic and cell-extrinsic factors, particularly the role of neural circuits and the activity of neurons that form synapses with the affected cells. A greater understanding of the role of circuit activity might substantially augment the outcome achieved by manipulating RGCs’ intrinsic growth potential and cell-extrinsic factors.

## Author Contributions

All authors conceptualized the study and wrote the manuscript.

## Conflict of Interest

The authors declare that the research was conducted in the absence of any commercial or financial relationships that could be construed as a potential conflict of interest.
